# Correction: Photodynamic antimicrobial chemotherapy with cationic phthalocyanines against *Escherichia coli* planktonic and biofilm cultures

**DOI:** 10.1039/d2ra90106d

**Published:** 2022-10-31

**Authors:** Min Li, Bingjie Mai, Ao Wang, Yiru Gao, Xiaobing Wang, Xin Liu, Shanshan Song, Quanghong Liu, Shaohua Wei, Pan Wang

**Affiliations:** Key Laboratory of Medicinal Resources and Natural Pharmaceutical Chemistry, Ministry of Education, National Engineering Laboratory for Resource Developing of Endangered Chinese Crude Drugs in Northwest of China, College of Life Sciences, Shaanxi Normal University Xi’an 710062 China wangpan@snnu.edu.cn +86-29-8531-0275; School of Chemistry and Materials Science, Jiangsu Key Laboratory of Biofunctional Materials, Jiangsu Collaborative Innovation Centre of Biomedical Functional Materials, Key Laboratory of Applied Photochemistry, Nanjing Normal University Wenyuan Road No. 1 Nanjing 210023 China shwei@njnu.edu.cn; Institute of Chemical Industry of Forest Products, Chinese Academy of Forestry No. 16, Suojin 5th Village Nanjing 210042 China

## Abstract

Correction for ‘Photodynamic antimicrobial chemotherapy with cationic phthalocyanines against *Escherichia coli* planktonic and biofilm cultures’ by Min Li *et al.*, *RSC Adv.*, 2017, **7**, 40734–40744, https://doi.org/10.1039/C7RA06073D.

The authors regret that incorrect versions of Fig. 7F (Control) and Fig. 8A (Light-alone) were included in the original article. The corrected versions are shown below. The correction does not change any results or conclusions of the original paper.

**Fig. 7:**
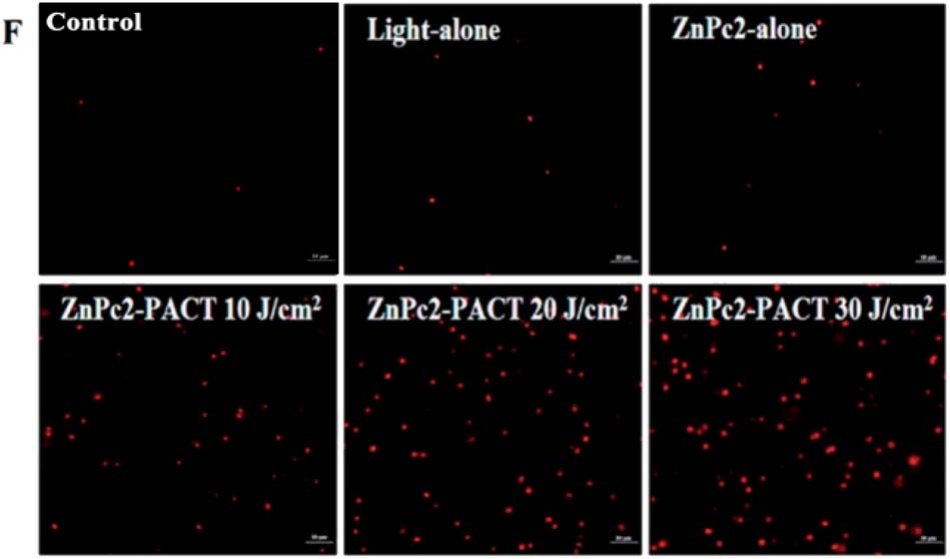
Membrane integrity detected by PI staining. (F) Images taken by fluorescence microscope of *E. coli* treated with 5 μM ZnPc2 in different groups.

**Fig. 8:**
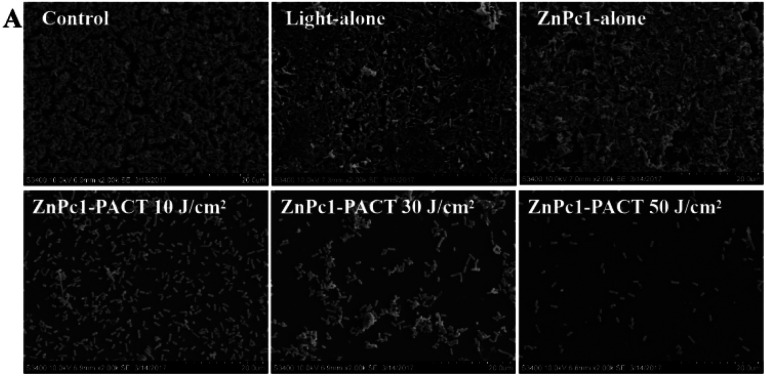
SEM images of PACT-subjected *E. coli* biofilms. (A) Images of *E. coli* treated with 20 μM ZnPc1-PACT in different groups.

The Royal Society of Chemistry apologises for these errors and any consequent inconvenience to authors and readers.

## Supplementary Material

